# Supplementary material for: The causal relationship between the human gut microbiota and pyogenic arthritis: a Mendelian randomization study
Source: Front Cell Infect Microbiol. 2024 Nov 26;14:1452480. doi: 10.3389/fcimb.2024.1452480 (PMC11629706; doi:10.3389/fcimb.2024.1452480)
Supplement: Supplementary file 1 [file DataSheet1.zip › mendelian test/ebi-a-GCST90027533.csv_leave_one_out.pdf]

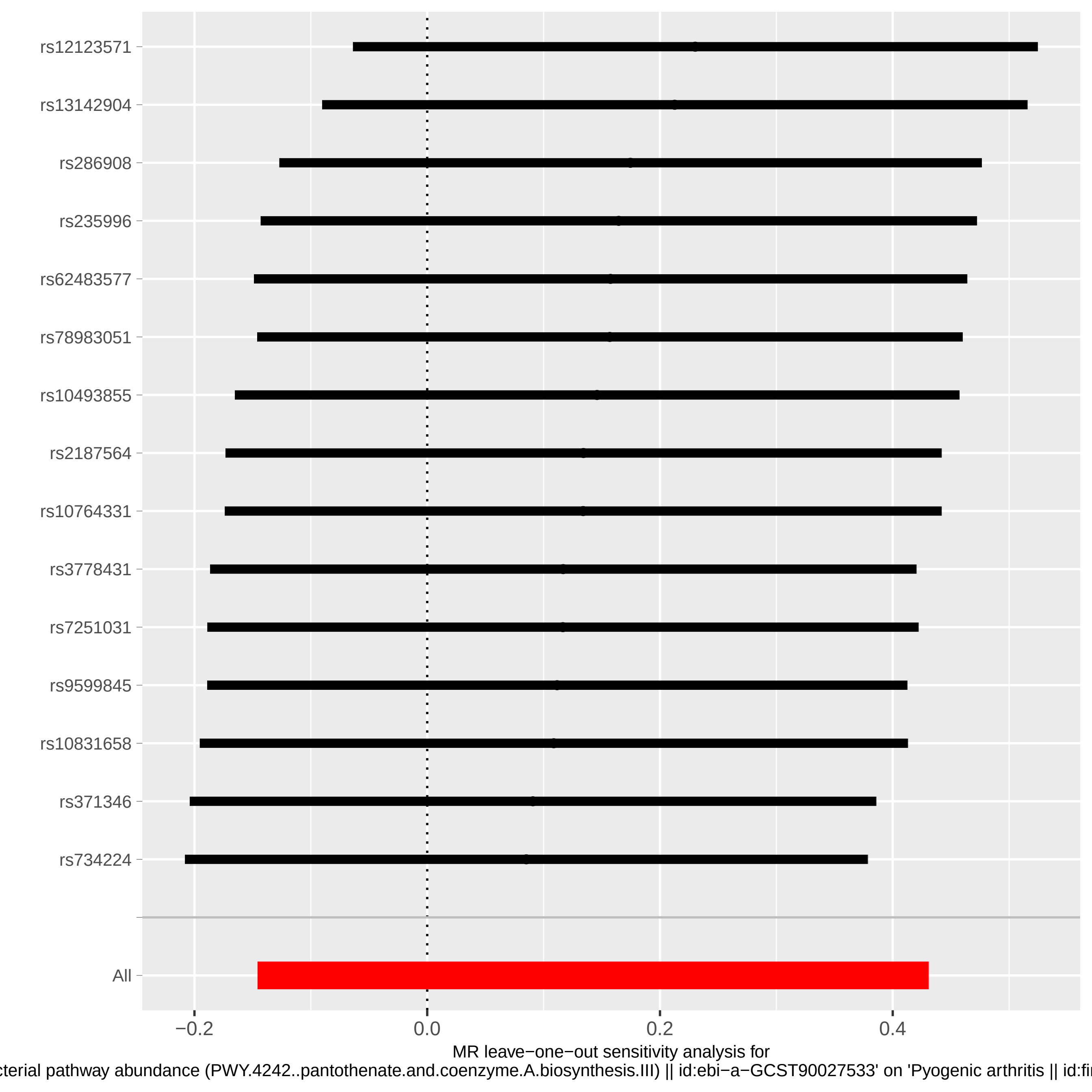

rs12123571
rs13142904
rs286908
rs235996
rs62483577
rs78983051
rs10493855
rs2187564
rs10764331
rs3778431
rs7251031
rs9599845
rs10831658
rs371346
rs734224
All
−0.2
0.0
0.2
0.4
MR leave−one−out sensitivity analysis for
cterial pathway abundance (PWY.4242..pantothenate.and.coenzyme.A.biosynthesis.III) || id:ebi−a−GCST90027533' on 'Pyogenic arthritis || id:fi
